# Serum Metrnl levels are decreased in subjects with overweight or obesity and are independently associated with adverse lipid profile

**DOI:** 10.3389/fendo.2022.938341

**Published:** 2022-09-05

**Authors:** Xiaoyu Ding, Xiaona Chang, Jiaxuan Wang, Nannan Bian, Yu An, Guang Wang, Jia Liu

**Affiliations:** Department of Endocrinology, Beijing Chao-yang Hospital, Capital Medical University, Beijing, China

**Keywords:** obesity, meteorin-like, adipose tissue, dyslipidemia, lipid metabolism

## Abstract

**Background:**

Meteorin-like (Metrnl), a novel adipokine, is highly expressed in adipose tissue and has a beneficial effect on energy metabolism. However, data on circulating Metrnl levels in obesity are scarce and inconsistent. This study aimed to evaluate the serum levels of Metrnl in adults with obesity and its association with glucose and lipid metabolism.

**Methods:**

182 subjects were included in the cross-sectional study. The participants were divided into three groups according to BMI: normal (n = 95), overweight (n = 46), and obesity (n = 41). Serum Metrnl concentrations were measured by enzyme-linked immunosorbent assay.

**Results:**

Serum Metrnl levels in overweight or obese subjects were significantly lower than in the normal group. Circulating Metrnl levels were negatively correlated with TG, TC, LDL-C, and sdLDL and positively correlated with HDL-C before and after adjusting for age, sex, BMI, diabetes, HOMA-IR, and eGFR (all *P* < 0.05). Furthermore, logistic regression analysis indicated that compared with the highest tertile, the lowest tertile of Metrnl levels were significantly associated with the presence of hyper-TG, hyper-TC, and Hyper-LDL after full adjustment (all *P* for trend < 0.05).

**Conclusions:**

Serum Metrnl levels were reduced in individuals with overweight or obesity and were independently associated with adverse lipid profile, suggesting that modifying circulating Metrnl levels may serve as a potential therapeutic target for atherogenic dyslipidemia.

## Introduction

Obesity has emerged as a worldwide public health issue, leading to a concomitant increase in associated complications such as insulin resistance, dyslipidemia, type II diabetes (T2DM), metabolic syndrome, fatty liver disease, cardiovascular disease (CVD), and cancer (1, 2). The root cause of obesity development is chronic caloric excess (3). Moreover, the massive expansion of adipose tissue results in lipid overflow and accumulation in visceral tissues, directly and indirectly contributing to metabolic disorders (4). Aside from serving as a major site of energy storage, adipose tissue also acts as an endocrine organ that regulates diverse biological processes, including glycolipid metabolism, angiogenesis, and inflammation (5, 6). Multiple secretory peptides and proteins (adipokines) derived from adipose tissue, such as adiponectin and leptin, have been identified, and dysregulated production and function of these adipokines are related to whole-body metabolic diseases (7, 8). Nevertheless, the metabolic actions of several new adipokines in obesity-linked metabolic disorders are still largely unknown.

Meteorin-like (Metrnl), also known as Subfatin, has been identified as a novel adipokine secreted by adipose tissue and skeletal muscle (9–11). Li et al. reported that Metrnl was highly expressed in both rodent and human adipose tissue (9). Acute cold exposure and exercise can induce Metrnl expression in white adipose tissue. Furthermore, increased circulating Metrnl levels promote energy expenditure and improve glucose tolerance in mice (10). In addition to inducing thermogenesis-related genes expression in beige/brown adipose tissue, Metrnl also can regulate adipocyte differentiation, lipid-mediated inflammation and insulin resistance (12, 13). However, data on circulating Metrnl concentrations in obesity and T2DM are conflicting (14–18). Thus, the present study aimed to evaluate the serum levels of Metrnl in individuals with obesity and its association with glucose and lipid metabolism.

## Materials and methods

### Study population

This cross-sectional study recruited participants (20-70 years) who underwent routine health checkups at the health medical center of Beijing Chao-yang Hospital Affiliated to Capital Medical University from June 2020 to December 2021. The exclusion criteria included type 1 diabetes mellitus, breastfeeding or pregnant, a history of stroke, myocardial infarction, heart failure, liver or kidney disease, thyroid dysfunction, cancer, chronic inflammation, acute infection, anemia, autoimmune diseases and use of any drugs that influence blood glucose and lipid, such as antidiabetics, statins, corticosteroids, and estrogen. At last, 182 participants were included in the final analysis. Written informed consent was obtained from all participants, and the study protocol was approved by the Ethics Committee of our hospital.

### Anthropometric and biochemical measurements

Height and body weight were measured as per standardized protocols. Body Mass Index (BMI) was calculated as weight divided by height squared (kg/m^2^). All venous blood samples were collected in the morning after an overnight fast. Total cholesterol (TC), triglyceride (TG), high-density lipoprotein cholesterol (HDL-C), low-density lipoprotein cholesterol (LDL-C), small dense low-density lipoprotein (sdLDL), and fasting blood glucose (FBG) were determined by an autoanalyzer (Hitachi 747, Roche Diagnostics, Germany). Fasting insulin (FINS) was detected by the chemiluminescence method (Dimension Vista, Siemens Healthcare Diagnostics). Hemoglobin A1c (HbA1c) was assessed by high-performance liquid chromatography (Tokyo, Japan). Serum Metrnl levels were measured using ELISA kits (R&D Systems, Minneapolis, MN, USA). The homeostasis model assessment–insulin resistance (HOMA-IR) was calculated as follows: FBG (mmol/L) × FINS (mIU/L)/22.5 (19). The estimated glomerular filtration rate (eGFR) was calculated as previously described (20).

### Definitions

All subjects were classified as three groups according to Chinese obesity working group definitions: normal (BMI < 24 kg/m^2^), overweight (BMI 24-27.9 kg/m^2^), and obesity (BMI ≥ 28 kg/m^2^) (21). The atherogenic lipid profiles: hypertriglyceridemia (hyper-TG) was defined as TG ≥ 1.7 mmol/L, hypercholesterolemia (hyper-TC) as TC ≥ 5.2 mmol/L, hypo-HDL cholesterolemia (hypo-HDL) as HDL-C < 1.0 mmol/L, and hyper-LDL cholesterolemia (hyper-LDL) as LDL-C ≥ 3.4 mmol/L (22). Diabetes was defined as FBG ≥ 7.0 mmol/L, HbA1c ≥ 6.5%, or self-reported previous diagnosis of diabetes (23).

### Statistical analysis

All analyses were performed using the IBM SPSS 26.0 (IBM Corp., Armonk, New York, USA) and GraphPad Prism 9.0 (Inc, CA, USA). The Kolmogorov–Smirnov test was conducted to assess the distribution of continuous variables. Data were expressed as mean ± standard deviation or median (interquartile range) for continuous variables. Data of categorical variables were expressed as number (%), and the Chi-square test was used to compare groups. Intergroup comparisons were performed using one-way ANOVA or Kruskal–Wallis test with the Bonferroni *post hoc* test. Additionally, a general linear model with one-way analysis of covariance (ANCOVA) followed by Bonferroni *post hoc* test was used to compare intergroup differences after adjusting for potential confounding factors. Spearman and partial correlation analysis were conducted to evaluate the correlation between serum Metrnl levels and metabolic parameters. To further detect the association of serum Metrnl levels with atherogenic dyslipidemia, linear regression and logistic regression models were performed. The variables that were considered clinically relevant or showed a significant relationship in correlation analyses, as well as that without collinearity were selected into logistic regression models. Model 1 was without adjustment; Model 2 was adjusted for age and sex; and Model 3 was further adjusted for BMI, diabetes, HOMA-IR, and eGFR. Receiver operating characteristic curve (ROC) analysis was carried out to determine the cut-off value of Metrnl. *P* < 0.05 (two-tailed) was considered statistically significant.

## Results

### Characteristics of study participants

The clinical characteristics of the study subjects are presented in **Table 1**. The participants were divided into three groups based on BMI. There were no statistically significant differences in terms of age and sex among the studied groups. Compared with the normal group, participants with overweight or obesity tended to have higher levels of TG, sdLDL, FBG, HbA1c, FINS, and HOMA-IR, while lower levels of HDL-C and serum Metrnl (all *P* < 0.05). However, there were no significant differences in TC, LDL-C, eGFR, and the proportion of diabetes among the three groups. Moreover, after adjusting for age, sex, and diabetes, serum Metrnl levels remained significantly lower in subjects with overweight or obesity (**Supplementary Table 1** **).** We also divided the subjects into three groups according to the tertiles of circulating Metrnl levels (**Table 2**). We observed that individuals with the lowest concentrations of Metrnl had higher TG (*P* < 0.001), TC (*P* = 0.006), LDL-C (*P* = 0.006), sdLDL (*P* < 0.001), FINS (*P* = 0.011), and HOMA-IR (*P* = 0.008) levels but lower HDL-C levels (*P* = 0.032).

**Table 1:** Clinical and biochemical characteristics of all the study participants.

Variable	Normal	Overweight	Obesity	*P*
	n = 95	n = 46	n = 41	
Sex, male, n (%)	15 (15.8)	13 (28.3)	11 (26.8)	0.151
Age, years	42.21 ± 10.77	45.50 ± 11.85	41.71 ± 12.07	0.100
BMI, kg/m^2^	21.51 ± 1.79	25.91 ± 1.17^***^	30.55 ± 2.41^***,###^	**< 0.001**
TG, mmol/L	0.86 (0.68, 1.23)	1.39 (1.07, 2.53)^***^	1.39 (1.09, 2.47)^***^	**< 0.001**
TC, mmol/L	5.14 ± 0.86	5.05 ± 0.97	5.02 ± 1.01	0.720
HDL-C, mmol/L	1.55 ± 0.34	1.32 ± 0.35^***^	1.14 ± 0.22^***,#^	**< 0.001**
LDL-C, mmol/L	3.19 ± 0.84	3.21 ± 0.90	3.48 ± 0.96	0.209
sdLDL, mmol/L	0.58 (0.43, 0.96)	0.93 (0.65, 1.24)^**^	1.02 (0.70, 1.32)^***^	**< 0.001**
FBG, mmol/L	4.89 (4.50, 5.20)	5.13 (4.76, 5.85)^**^	5.30 (5.00, 5.80)^***^	**< 0.001**
HbA1c, %	5.5 (5.2, 5.6)	5.6 (5.3, 6.1)	5.7 (5.4, 6.0)^*^	**0.012**
FINS, uIU/mL	7.00 (4.85, 9.23)	12.15 (9.30, 17.20)^***^	17.50 (12.90, 24.30)^***^	**< 0.001**
HOMA-IR	1.45 (0.98, 2.08)	2.82 (2.16, 4.30)^***^	4.40 (3.22, 6.24)^***,#^	**< 0.001**
Diabetes, n (%)	2 (2.1)	5 (10.9)	4 (9.8)	0.065
eGFR, mL/min/1.73 m^2^	111.16 ± 12.92	108.68 ± 12.53	109.28 ± 16.30	0.545
Metrnl, pg/ml	221.6 (191.6, 250.5)	195.9 (167.4, 215.5)^**^	183.7 (156.8, 227.7)^***^	**< 0.001**

Data were expressed as the mean ± SD or median (interquartile range) unless stated otherwise. P values for categorical variables were calculated using Chi-square test, and P values for continuous variables were calculated using one-way ANOVA test or Kruskal–Wallis test with the Bonferroni post hoc test. Bold indicates P value < 0.05. *Compared with normal; ^#^ Compared with overweight. *,^#^ P < 0.05; **,^##^ P < 0.01; ***,^###^ P < 0.001. BMI, body mass index; TG, triglycerides; TC, total cholesterol; HDL-C, high-density lipoprotein cholesterol; LDL-C, low-density lipoprotein cholestero; FBG, fasting blood glucose; FINS, fasting insulin; HOMA-IR, homeostasis model assessment–insulin resistance; eGFR, estimated glomerular filtration rate; Metrnl, Meteorin-like.

**Table 2:** Clinical characteristics of the study population according to the tertiles of serum Mternl levels.

Variable	Tertile 1	Tertile 2	Tertile 3	*P*
	n = 60	n = 61	n = 61	
Sex, male, n (%)	11 (18.3)	10 (16.4)	18 (29.5)	0.163
Age, years	42.08 ± 10.15	43.87 ± 10.19	42.82 ± 13.58	0.689
BMI, kg/m^2^	26.25 ± 4.66	24.19 ± 3.29^*^	23.56 ± 3.81^**^	**0.001**
TG, mmol/L	1.47 (1.06, 2.54)	1.05 (0.78, 1.85)^*^	0.88 (0.71, 1.21)^***,#^	**< 0.001**
TC, mmol/L	5.37 ± 0.91	5.07 ± 0.94	4.84 ± 0.85^**^	**0.006**
HDL-C, mmol/L	1.31 ± 0.31	1.41 ± 0.37	1.48 ± 0.38^*^	**0.032**
LDL-C, mmol/L	3.55 ± 0.88	3.20 ± 0.91	3.04 ± 0.82^**^	**0.006**
sdLDL, mmol/L	1.00 (0.69, 1.39)	0.79 (0.46, 1.09)^**^	0.59 (0.44, 0.95)^***^	**< 0.001**
FBG, mmol/L	5.09 (4.77, 5.53)	4.98 (4.61, 5.32)	5.12 (4.73, 5.42)	0.178
HbA1c, %	5.6 (5.3, 5.9)	5.5 (5.2, 5.7)	5.5 (5.2, 5.8)	0.194
FINS, uIU/mL	11.5 (8.20, 18.25)	9.95 (6.35, 14.70)	8.20 (5.80, 11.35)^**^	**0.011**
HOMA-IR	2.62 (1.82, 5.05)	2.28 (1.30, 3.41)	1.76 (1.24, 2.69)^**^	**0.008**
Diabetes, n (%)	5 (8.3)	4 (6.6)	2 (3.3)	0.496
eGFR, mL/min/1.73 m^2^	113.08 ± 12.23	109.90 ± 11.53	107.40 ± 16.25	0.071
Metrnl, pg/ml	168.3(152.2, 181.0)	205.5 (198.2, 215.0)^***^	250.5 (235.3, 291.0)^***,###^	**< 0.001**

Data were expressed as the mean ± SD or median (interquartile range) unless stated otherwise. P values for categorical variables were calculated using Chi-square test, and P values for continuous variables were calculated using one-way ANOVA test or Kruskal–Wallis test with the Bonferroni post hoc test. Bold indicates P value < 0.05. *Compared with tertile 1; ^#^ Compared with tertile 2. *,^#^ P < 0.05; **,^##^ P < 0.01; ***,^###^ P < 0.001. BMI, body mass index; TG, triglycerides; TC, total cholesterol; HDL-C, high-density lipoprotein cholesterol; LDL-C, low-density lipoprotein cholestero; FBG, fasting blood glucose; FINS, fasting insulin; HOMA-IR, homeostasis model assessment–insulin resistance; eGFR, estimated glomerular filtration rate; Metrnl, Meteorin-like.

### Correlation of serum Metrnl levels with clinical parameters

**Figure 1:**
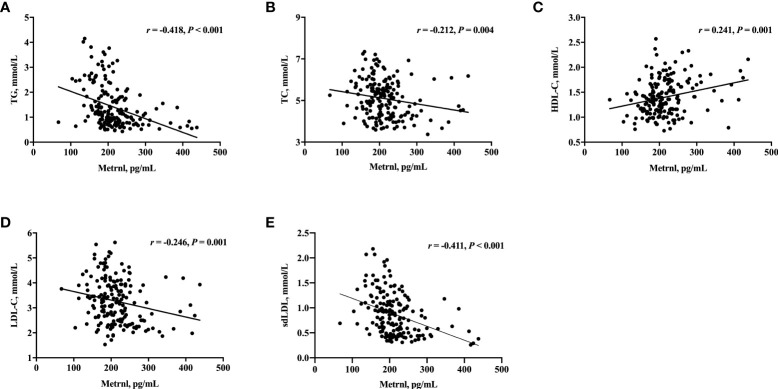
Correlation of serum Metrnl levels with TG **(A)**, TC **(B)**, HDL-C **(C)**, LDL-C **(D)**, and sdLDL **(E)** in 182 subjects. Spearman’s correlation was performed for analysis due to the skewed distribution of Metrnl. *P* values < 0.05 were considered statistically significant.

We next evaluated the relationship of serum Metrnl levels with metabolic parameters in all subjects using correlation analysis (**Table 3**, **Figure 1**). Circulating Metrnl concentrations were negatively correlated with BMI (*r* = −0.297, *P* < 0.001), TG (*r* = −0.418, *P* < 0.001), TC (*r* = −0.212, *P* = 0.004), LDL-C (*r* = −0.246, *P* = 0.001), sdLDL (*r* = −0.411, *P* < 0.001), FINS (*r* = −0.301, *P* < 0.001), HOMA-IR (*r* = −0.301, *P* < 0.001), and eGFR (*r* = −0.153, *P* = 0.040), and were positively associated with HDL-C (*r* = 0.241, *P* = 0.001). Of note, after adjusting for age, sex, BMI, and eGFR, the correlations of serum Metrnl levels with TG, TC, LDL-C, HDL-C, and sdLDL remained statistically significant, except for FINS and HOMA-IR.

**Table 3:** Correlation analysis between serum Mternl levels and clinical variables.

	Metrnl		Metrnl*	
	*r*	*P*	*r*	*P*
Age	-0.002	0.976		
BMI	-0.297	**< 0.001**		
TG	-0.418	**< 0.001**	-0.380	**< 0.001**
TC	-0.212	**0.004**	-0.262	**0.001**
HDL-C	0.241	**0.001**	0.237	**0.003**
LDL-C	-0.246	**0.001**	-0.263	**0.001**
sdLDL	-0.411	**< 0.001**	-0.386	**< 0.001**
FBG	-0.104	0.162	-0.056	0.491
HbA1c	-0.143	0.084	-0.049	0.561
FINS	-0.301	**< 0.001**	-0.103	0.204
HOMA-IR	-0.301	**< 0.001**	-0.112	0.157
eGFR	-0.153	**0.040**		

P values were performed by Spearman’s correlation analysis and *partial correlation analysis adjustment for age, sex, BMI, and eGFR. Bold indicates P value < 0.05.

ANCOVA showed that TG, TC, LDL-C, and sdLDL levels were progressively decreased across serum Metrnl tertiles after adjusting for age, sex, BMI, and diabetes (all *P* < 0.01, **Supplementary Table 2**). In addition, further linear regression analysis indicated that lower circulating Metrnl levels were significantly associated with higher TG, TC, LDL-C, and sdLDL as well as lower HDL-C, even after full adjustment (**Supplementary Table 3**).

### Serum Metrnl levels are independently associated with adverse lipid metabolism

**Figure 2:**
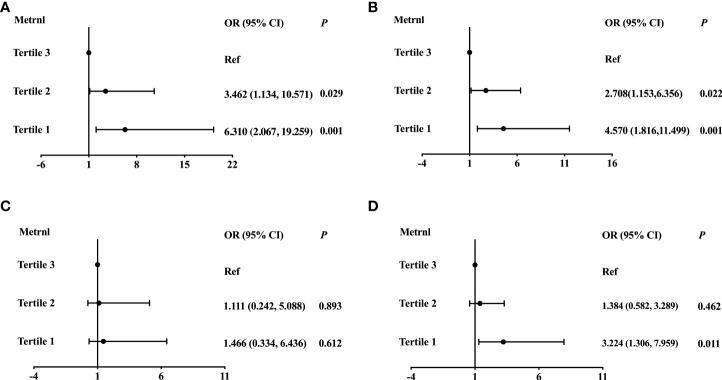
Logistic regression analysis of the association between serum Mternl levels with Hyper-TG **(A)**, Hyper-TC **(B)**, Hypo-HDL **(C)**, and Hyper-LDL **(D)**. Adjusted for age, sex, BMI, diabetes, HOMA-IR, and eGFR.

To further determine whether serum Metrnl levels were independently associated with lipid metabolism, we conducted logistic regression analysis in all participants (**Table 4**). In unadjusted logistic regression model, compared with the highest tertile, lower serum Metrnl levels were significantly associated with the presence of hyper-TG [OR (95%CI), 6.312 (2.472-16.115)] and hyper-TC [2.891 (1.373-6.086)] (all *P* for trend < 0.05), although not with Hypo-HDL, and these associations remained significant even after adjustment for confounding factors (model 2 and model 3). In addition, after further adjusting for age, sex, BMI, diabetes, HOMA-IR, and eGFR in model 3, a lower serum Metrnl level was significantly associated with an increased risk of hyper-LDL [3.224 (1.306-7.959), *P* = 0.011] (**Figure 2**). Meanwhile, per 1-unit decreased circulating Metrnl levels were significantly correlated with the presence of hyper-TG, hyper-TC, and hyper-LDL both before and after adjusting for potential confounders, respectively.

**Table 4:** Logistic regression analysis of the association between serum Mternl levels and adverse lipid profile.

OR (95% CI)	Per rank decrease			*P* for trend	Per 1-unit decrease	*P*
	Tertile 3	Tertile 2	Tertile 1			
Hyper-TG						
Model 1	1.00 (Ref)	2.981 (1.134, 7.831)	6.312 (2.472, 16.115)	**0.001**	1.019 (1.010, 1.029)	**< 0.001**
Model 2	1.00 (Ref)	3.734 (1.356, 10.280)	8.052 (2.974, 21.800)	**< 0.001**	1.020 (1.011, 1.031)	**< 0.001**
Model 3	1.00 (Ref)	3.462 (1.134, 10.571)	6.310 (2.067, 19.259)	**0.005**	1.022 (1.010, 1.034)	**< 0.001**
Hyper-TC						
Model 1	1.00 (Ref)	2.139 (1.022, 4.478)	2.891 (1.373, 6.086)	**0.017**	1.007 (1.002, 1.013)	**0.011**
Model 2	1.00 (Ref)	2.218 (1.037, 4.743)	3.147 (1.462, 6.775)	**0.012**	1.008 (1.002, 1.014)	**0.008**
Model 3	1.00 (Ref)	2.708 (1.153, 6.356)	4.570 (1.816, 11.499)	**0.005**	1.010 (1.003, 1.017)	**0.005**
Hypo-HDL						
Model 1	1.00 (Ref)	0.672 (0.250, 1.810)	0.486 (0.167, 1.412)	0.400	1.004 (0.996, 1.013)	0.289
Model 2	1.00 (Ref)	0.907 (0.283, 2.912)	1.701 (0.604, 4.789)	0.448	1.005 (0.997, 1.013)	0.229
Model 3	1.00 (Ref)	1.111 (0.242, 5.088)	1.466 (0.334, 6.436)	0.865	1.004 (0.992, 1.015)	0.494
Hyper-LDL						
Model 1	1.00 (Ref)	0.723 (0.235, 2.223)	1.487 (0.553, 4.003)	0.109	1.006 (1.001, 1.012)	**0.028**
Model 2	1.00 (Ref)	1.158 (0.543, 2.469)	2.269 (1.070, 4.808)	0.070	1.007 (1.001, 1.013)	**0.016**
Model 3	1.00 (Ref)	1.384 (0.582, 3.289)	3.224 (1.306, 7.959)	**0.029**	1.009 (1.001, 1.016)	**0.019**

Model 1: unadjusted; Model 2: adjusted for age and sex; Model 3: adjusted for age, sex, BMI, diabetes, HOMA-IR, and eGFR. Bold indicates P value < 0.05.

**Figure 3:**
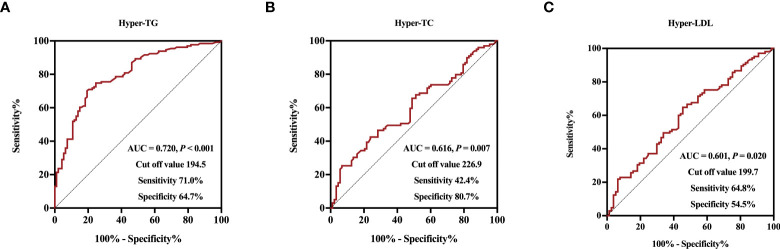
Receiver operative characteristic curves and cut off values of serum Metrnl levels for Hyper-TG **(A)**, Hyper-TC **(B)**, and Hyper-LDL **(C)**.

Finally, we performed ROC analysis to identify the cut off value of Metrnl for discriminating adverse lipid profile (**Figure 3**). The optimal cut off values of circulating Metrnl for discriminating hyper-TG, hyper-TC, and hyper-LDL were 194.5 pg/mL (area under curve, AUC = 0.720, *P* < 0.001), 226.9 pg/mL (AUC = 0.616, *P* = 0.007), and 199.7 pg/mL (AUC = 0.601, *P* = 0.020), respectively.

## Discussion

Adipokines are involved in the intra- and inter-tissue communication and play an essential modulatory role in the maintenance of whole-body metabolic homeostasis. However, under certain pathological conditions, such as obesity, dysregulated biosynthesis and secretion of adipokines contributes to the development of various metabolic disorders. In this study, we observed that serum Metrnl concentrations were decreased in subjects with overweight or obesity. More strikingly, lower circulating Metrnl levels were independently associated with adverse lipid profile. These findings suggest that Metrnl may be a promising therapeutic target for atherogenic dyslipidemia.

Metrnl, a novel-secreted protein, is mainly produced by adipose tissue and muscle. As a novel adipokine, it has been shown to increase insulin sensitivity through the peroxisome proliferator-activated receptor-gamma (PPARγ) pathway (10, 12). Although several studies have reported the correlation between circulating levels of Metrnl and T2DM, the results are inconsistent. A recent meta-analysis showed no significant association between circulating Metrnl levels and T2DM, due to uncontrolled confounding factors (24). Furthermore, some studies showed that circulating Metrnl levels reduced in patients with T2DM and negatively correlated with insulin resistance (16, 25, 26). However, Chung et al. have demonstrated that circulating Metrnl concentrations were elevated in patients with T2DM compared to controls, and similar results were reported in another study (14, 27). In our study, we observed serum Metrnl levels were inversely related to HOMA-IR, whereas this relationship was attenuated and no longer significant after adjusting for age, sex, BMI, and eGFR. One explanation could be that obesity may significantly increase insulin resistance, in correlation analyses that are not adjusted for BMI, the “true” association between serum Metrnl levels and HOMA-IR is confounded. After these strong confounding variables had been considered, the “true” association between them became disappear.

Previous studies have indicated that lower circulating Metrnl concentrations are linked to obesity (18, 28). Consistently, we also observed that serum Metrnl levels were decreased in adults with overweight or obesity and were significantly related to BMI. Nevertheless, other studies have reported opposite results. Wang et al. noticed that serum Metrnl levels were higher in overweight and obese subjects than in normal weight subjects. Moreover, El-Ashmawy et al. showed that circulating Metrnl values were not correlated with BMI (29). The discrepancy might partly attribute to ethnic difference, sample size, concomitant disease, or the heterogeneity in study design. Interestingly, a recent study reported that serum Metrnl levels were significantly lower in obese patients with osteoarthritis compared to obese subjects without osteoarthritis, whereas an opposite pattern was found when assessing Metrnl levels in synovial fluid, suggesting different independent regulatory mechanism for Metrnl production from various tissues (30). In addition, it has been shown that systemic Metrnl is mainly secreted by adipose tissue and muscle (9, 10). Obesity may increase the loss of muscle mass, so we speculated that the observed lower circulating Metrnl levels in obesity might result from adipose tissue dysfunction and sarcopenia. Further research is warranted to better elucidate the role of circulating Metrnl in obesity.

Weight gain and adipose tissue dysfunction often have been linked to atherogenic lipid profile, characterized by increased circulating levels of TG, LDL-C, and sdLDL, as well as decreased levels of HDL-C (31). This atherogenic dyslipidemia is a major risk factor for the development of CVD (32). It is noteworthy here that serum Metrnl levels were significantly correlated with TG, TC, LDL-C, HDL-C, and sdLDL, even after adjustment for age, sex, BMI, diabetes, HOMA-IR, and eGFR. Additionally, logistic regression analysis revealed that reduced circulating Metrnl levels were independently associated with increased risk of hyper-TG, hyper-TC, and hyper-LDL in the current study. Sparklingly, results from clinical studies have revealed that decreased systemic Metrnl levels are related to coronary artery disease and chronic heart failure (33, 34). Besides, very recently, it has been indicated that the lack of Metrnl is prone to promote cardiac hypertrophy, and the overexpression or treatment of Metrnl can activate the PPARγ coactivator-1α (PGC1-α) and fatty acid oxidation (FAO) pathways of cardiomyocytes to protect heart against cardiac dysfunction in mice (35).

Apart from its protective effect on the heart, circulating Metrnl also has beneficial effects on adipose tissue and skeletal muscle. An increase in circulating Metrnl can stimulate whole-body energy expenditure by inducing a broad beige/brown fat thermogenic gene (10). Notably, adipocyte Metrnl can also involve in TG metabolism. Deficiency of adipose tissue Metrnl exacerbated high fat diet induced hypertriglyceridemia, whereas adipose tissue-specific overexpression of Metrnl attenuated hypertriglyceridemia and insulin resistance in mice through PPARγ signaling (12). Meanwhile, Metrnl upregulated lipid metabolism-related genes and enhanced lipase activity in adipose tissue, suggesting an important role of Metrnl in adipose lipid metabolism (11). Treatment with Metrnl alleviated lipid-induced inflammation and induced FAO by AMPK or PPARγ signaling in skeletal muscle (13). In addition, tissue-specific Metrnl has been identified to control different components of blood lipids in mice (36). Hence, there is a possible explanation for the association between decreased circulating Metrnl levels and adverse lipid profile: Reduced levels of circulating Metrnl can decrease FAO by inhibiting lipoprotein lipase and lead to increased TG production in adipose tissue and liver, thereby contributing to hypertriglyceridemia, which further triggers impaired cholesteryl esters (HDL-C, LDL-C, and sdLDL) metabolism. Overall, further investigations are needed to explore the exact mechanism of Metrnl in regulating lipid metabolism.

Our study had several limitations. Firstly, the sample size of the study was relatively small, and the cross-sectional design could not establish a causal relationship between Metrnl and disorders. Secondly, body fat percentage, free fatty acids and calculated adipose tissue insulin resistance index were not measured, which might hamper the power of our study. Thirdly, it was not to exclude other potential confounders, especially exercise and cold exposure. Lastly, our study included only Chinese people, so the generalizability of our results might be a concern.

In conclusion, Serum Metrnl levels were decreased in individuals with overweight or obesity and were independently associated with adverse lipid profile. Our study indicates that modifying circulating Metrnl levels may be a promising therapeutic approach for atherogenic dyslipidemia.

## Data availability statement

The original contributions presented in this study are included in the article/**Supplementary Material**. Further inquiries can be directed to the corresponding authors.

## Ethics statement

The studies involving human participants were reviewed and approved by the Ethics Committee of Beijing Chao-yang Hospital Affiliated to Capital Medical University. The patients/participants provided their written informed consent to participate in this study.

## Author contributions

All authors contributed to the study conception and design. Material preparation, data collection and analysis were performed by XD, XC, JW, NB, and YA. The first draft of the manuscript was written by XD. The paper was revised by JL and GW. All authors contributed to the article and approved the submitted version.

## Funding

This study was supported by Beijing Natural Science Foundation Z20019 to LJ.

## Acknowledgments

The authors would like to thank all team members and participants in this study.

## Conflict of interest

The authors declare that the research was conducted in the absence of any commercial or financial relationships that could be construed as a potential conflict of interest.

## Publisher’s note

All claims expressed in this article are solely those of the authors and do not necessarily represent those of their affiliated organizations, or those of the publisher, the editors and the reviewers. Any product that may be evaluated in this article, or claim that may be made by its manufacturer, is not guaranteed or endorsed by the publisher.
