# Supplementary material for: Serum Metrnl levels are decreased in subjects with overweight or obesity and are independently associated with adverse lipid profile
Source: Front Endocrinol (Lausanne). 2022 Sep 5;13:938341. doi: 10.3389/fendo.2022.938341 (PMC9483104; doi:10.3389/fendo.2022.938341)
Supplement: Supplementary file 1 [file Table_1.docx]

# Supplementary Table 1. Serum Metrnl levels in subjects on the spectrum from normal weight to obesity.

|  | Normal | Overweight | Obesity | *P* |
| --- | --- | --- | --- | --- |
| Metrnl | 2.357 (2.336, 2.379) | 2.282 (2.251, 2.313)^***^ | 2.263 (2.230, 2.295)^***^ | **< 0.001** |

Data were expressed as adjusted mean and 95% confidence interval (CI). *P* values were performed by analysis of covariance (ANCOVA) adjusted for age, sex, and diabetes. Metrnl were log transformed for analysis. Bold indicates *P* value < 0.05. * Compared with tertile 1; # Compared with tertile 2. *, # *P* < 0.05; **, ## *P* < 0.01; ***, ### *P* < 0.001.
