# Supplementary material for: Serum Metrnl levels are decreased in subjects with overweight or obesity and are independently associated with adverse lipid profile
Source: Front Endocrinol (Lausanne). 2022 Sep 5;13:938341. doi: 10.3389/fendo.2022.938341 (PMC9483104; doi:10.3389/fendo.2022.938341)
Supplement: Supplementary file 2 [file Table_2.docx]

# Supplementary Table 2. Serum lipids levels across the tertiles of serum Metrnl levels.

|  | Tertile 1 | Tertile 2 | Tertile 3 | *P* |
| --- | --- | --- | --- | --- |
| TG | 0.179 (0.129, 0.230) | 0.091 (0.042, 0.140)^*^ | -0.024 (-0.073, 0.026)^***,##^ | **< 0.001** |
| TC | 5.430 (5.199, 5.660) | 5.047 (4.824, 5.270) | 4.797 (4.570, 5.024)^**^ | **0.001** |
| HDL-C | 1.370 (1.291, 1.450) | 1.373 (1.296, 1.450) | 1.458 (1.379, 1.536) | 0.221 |
| LDL-C | 3.551 (3.326, 3.776) | 3.217 (2.999, 3.434) | 3.026 (2.804, 3.247)^**^ | **0.006** |
| sdLDL | -0.014 (-0.06, 0.032) | -0.120 (-0.165, -0.076)^**^ | -0.200 (-0.249, -0.152)^***^ | **< 0.001** |

Data were expressed as adjusted mean and 95% confidence interval (CI). *P* values were performed by analysis of covariance (ANCOVA) adjusted for age, sex, BMI, and diabetes. Metrnl, TG, and sdLDL were log transformed for analysis. Bold indicates *P* value < 0.05. * Compared with tertile 1; # Compared with tertile 2. *, # *P* < 0.05; **, ## *P* < 0.01; ***, ### *P* < 0.001.
