# Supplementary material for: Serum Metrnl levels are decreased in subjects with overweight or obesity and are independently associated with adverse lipid profile
Source: Front Endocrinol (Lausanne). 2022 Sep 5;13:938341. doi: 10.3389/fendo.2022.938341 (PMC9483104; doi:10.3389/fendo.2022.938341)
Supplement: Supplementary file 3 [file Table_3.docx]

Supplementary Table 3. Linear regression analysis of the correlation between serum Mternl levels and serum lipids.

|  | Model 1 | | Model 2 | | Model 3 | |
| --- | --- | --- | --- | --- | --- | --- |
|  | Standardized *β* (*t*) | *P* | Standardized *β* (*t*) | *P* | Standardized *β* (*t*) | *P* |
| TG | -0.396 (-5.761) | **< 0.001** | -0.465 (-6.635) | **< 0.001** | -0.474 (-5.586) | **< 0.001** |
| TC | -0.188 (-2.565) | **0.011** | -0.205 (-2.761) | **0.006** | -0.271 (-3.716) | **< 0.001** |
| HDL-C | 0.251 (3.484) | **0.001** | 0.303 (4.036) | **< 0.001** | 0.223 (2.454) | **0.015** |
| LDL-C | -0.226 (-3.108) | **0.002** | -0.250 (-3.405) | **0.001** | -0.261 (-3.512) | **0.001** |
| sdLDL | -0.398 (-5.631) | **< 0.001** | -0.504 (-6.949) | **< 0.001** | -0.450 (-5.516) | **< 0.001** |

Model 1: unadjusted; Model 2: adjusted for age and sex; Model 3: adjusted for age, sex, BMI, diabetes, HOMA-IR, and eGFR. Metrnl, TG, and sdLDL were log transformed for analysis. Bold indicates *P* value < 0.05.
